# Insights into the Evolution of a Complex Virus from the Crystal Structure of Vaccinia Virus D13

**DOI:** 10.1016/j.str.2011.03.023

**Published:** 2011-07-13

**Authors:** Mohammad W. Bahar, Stephen C. Graham, David I. Stuart, Jonathan M. Grimes

**Affiliations:** 1 The Division of Structural Biology and the Oxford Protein Production Facility, Wellcome Trust Centre for Human Genetics, University of Oxford, Oxford, OX3 7BN, UK; 2 Science Division, Diamond Light Source Ltd., Diamond House, Harwell Science and Innovation Campus, Didcot, Oxfordshire OX11 0DE, UK

## Abstract

The morphogenesis of poxviruses such as vaccinia virus (VACV) sees the virion shape mature from spherical to brick-shaped. Trimeric capsomers of the VACV D13 protein form a transitory, stabilizing lattice on the surface of the initial spherical immature virus particle. The crystal structure of D13 reveals that this major scaffolding protein comprises a double β barrel “jelly-roll” subunit arranged as pseudo-hexagonal trimers. These structural features are characteristic of the major capsid proteins of a lineage of large icosahedral double-stranded DNA viruses including human adenovirus and the bacteriophages PRD1 and PM2. Structure-based phylogenetic analysis confirms that VACV belongs to this lineage, suggesting that (analogously to higher organism embryogenesis) early poxvirus morphogenesis reflects their evolution from a lineage of viruses sharing a common icosahedral ancestor.

## Highlights

► Poxvirus D13 acts as a scaffold for the morphogenesis of spherical immature virions ► D13 has a double “jelly-roll” structure, like other large DNA virus capsid proteins ► Structure-based phylogenetics places D13 into an icosahedral viral lineage ► Poxvirus morphogenesis reflects *Vaccinia virus* evolution from an icosahedral ancestor

## Introduction

*Vaccinia virus* (VACV), the smallpox vaccine, is the prototypic member of the family *Poxviridae* (Moss, 2007). These large, enveloped, double-stranded (ds) DNA viruses replicate and assemble in the cytoplasm of host cells via a complex morphogenic pathway that gives rise to a virion structure that lacks the helical or icosahedral symmetry of other viral capsids (Condit et al., 2006).

VACV morphogenesis begins in cytoplasmic “viral factories” and the first distinct structures to appear are crescent-shaped precursor membranes, bearing a honeycomb layer on their outer surface (Moss, 2007). These membrane crescents progressively expand and seal themselves to form the spherical immature virus (IV) particles, which contain the viral genome and core proteins. Deep-etch electron microscopy studies have shown that the growing crescent membranes and closed IV particles contain a single membrane bilayer (Heuser, 2005) stabilized by the honeycomb surface lattice formed by homotrimers of the ∼62 kDa D13 protein, assembled in a hexagonal mesh on the exterior of the IV membrane (Szajner et al., 2005). The D13 lattice seems to act as a mechanical scaffold for the growing crescent membrane of the IV with sufficient rigidity to maintain a constant radius of curvature of the crescents even before they close to form complete spheres (Chlanda et al., 2009; Heuser, 2005), thereby fixing the size of the immature virion.

IV particles undergo a major morphological change, condensing into the brick-shaped intracellular mature viruses (IMVs) that represent the majority of infectious progeny virus and remain inside cells until lysis. These morphogenic changes are poorly understood but result in a loss of the D13 lattice, which is not present in the mature forms of poxvirus virions (Essani et al., 1982; Heuser, 2005). A different paracrystalline coat is observed on the surface of IMV particles (Heuser, 2005; Spehner et al., 2004) and a number of core proteins undergo proteolytic cleavage, such as A10, A3, and L4 (Moss and Rosenblum, 1973; Sodeik and Krijnse-Locker, 2002) in a process coupled to the condensation of the viral core. A small percentage of IMV particles mature further by being wrapped in a double-membrane from early endosomes or the *trans*-Golgi network to form intracellular enveloped virus (IEV), then fusing with the cell membrane to exit the host cell as extracellular enveloped virus (EEV) (Smith et al., 2002).

A mutant of D13 with a single amino acid change from aspartic acid to glycine at position 513 (D13^D513G^) polymerizes into flat sheets of protein composed of hexagonal lattices (Szajner et al., 2005), which do not associate with membrane bilayers but form layered stacks (Szajner et al., 2005), and irregular membranes are formed that lack a D13 scaffold, similar to those seen when the expression of D13 is repressed (Zhang and Moss, 1992). These contrast with the curved honeycombs formed by wild-type D13, which contain both hexagonal and a limited number of pentagonal facets and associate tightly with IV membranes; demonstrating that D13 is crucial for defining the morphology of IV particles (Heuser, 2005). It is not known if the mutation abrogates the association of D13 with the IV membrane bilayer, or increases the self-association of D13 trimers.

When VACV infected cells are treated with the morphogenesis-inhibiting drug rifampicin (Moss et al., 1969) D13 does not associate with IV membranes, instead accumulating in large cytoplasmic inclusion bodies and irregular viral membranes are produced, similar to those seen in the absence of D13 expression (Moss et al., 1969). Removal of rifampicin reverses these effects (Sodeik et al., 1994). These and other studies suggest that D13 is the specific target of rifampicin.

D13 orthologs are present in all poxvirus family members, sharing a high degree of sequence conservation. Comparative genome analysis has suggested that there may be sequence similarities between D13 and the major capsid proteins (MCPs) of certain large eukaryotic dsDNA viruses (Iyer et al., 2001). This tentative similarity was found to be consistent with the low resolution electron microscopy (EM) structure of orfv075, the trimeric D13 ortholog from Orf virus (family *Poxviridae*) (Hyun et al., 2007), which allowed a plausible docking of the atomic structure of the VP54 capsid protein from *Paramecium bursaria Chlorella* virus type 1 (PBCV-1) (Hyun et al., 2007); consistent with D13 adopting the double β barrel fold seen in MCPs of the PRD1-adenovirus lineage of icosahedral dsDNA viruses (Abrescia et al., 2008; Bamford et al., 2005).

In order to better understand its role in morphogenesis and assembly we initiated structural studies of VACV D13. We report here the X-ray crystal structures of wild-type D13 and the mutant D13^D513G^ to resolutions of 2.8 Å and 3.0 Å, respectively. These analyses reveal the strong similarity of D13 to the major capsid proteins of dsDNA viruses such as adenovirus, PRD1 and PBCV-1, confirming that the major scaffolding protein of poxviruses contains a double β barrel “jelly-roll” structure. In addition, these structures suggest atomic details of D13 trimer interactions in the honeycomb lattice and implications for association with the nascent viral membranes. Collectively, these results place VACV into an existing structure-based phylogenetic lineage, and suggest that the morphogenesis of immature poxvirus virions reiterates their evolution from an ancient icosahedral ancestor.

## Results

### Structure Determination

VACV D13 and mutant D13^D513G^ with N-terminal hexahistidine tags were expressed in *Escherichia coli* and purified by Ni^2+^-affinity and size-exclusion chromatography as trimers. Crystals for native and SeMet forms of both proteins were isomorphous and belonged to space group *P*6_1_22, with one trimer in the crystallographic asymmetric unit. The structure of D13^D513G^ was solved by SAD analysis of SeMet-labeled protein crystals, and refined to 3.0 Å resolution with residuals *R*/*R*_free_ = 0.170/0.203. The structure of native wild-type D13 was solved from the phases provided by the isomorphous SAD structure of D13^D513G^, and refined to 2.8 Å with *R*/*R*_free_ = 0.177/0.206. In both structures, continuous electron density is present from residues Ser15 to Met547; the final four residues (548–551) being disordered. For the structure of D13^D513G^ at 3.0 Å, density has been attributed to residues Asn2 to Ile10 and modeled (no density is observed between Ile10 and Ser15). The N-terminal hexahistidine tag is disordered in both structures.

### Subunit Structure

**Figure 1:**
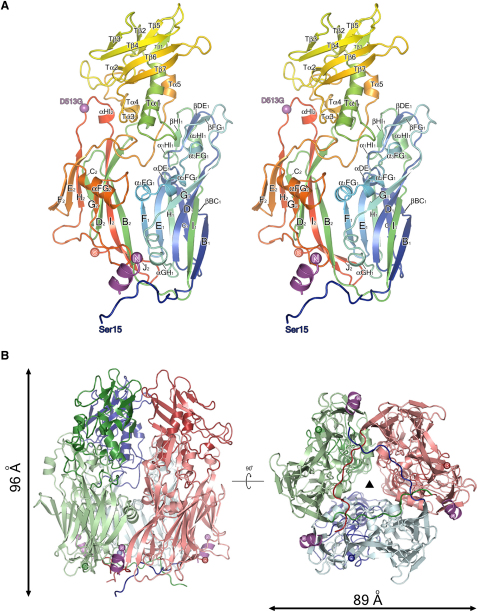
Structure of VACV D13 (A) Stereo ribbon representation of a monomer of D13 colored from blue (N terminus) to red (C terminus). The turret is treated as a separate domain with α helices and β strands labeled with prefix “T” for clarity. The N-terminal helix present in D13^D513G^ is shown in magenta. The position of the D513G mutation is shown with a sphere, colored magenta, and labeled. (B) Structure of the D13 trimer viewed from the side (left) and from below (right). Each subunit is colored separately, with N-terminal tails and turret domains being a darker shade compared to the jelly-rolls. Approximate molecular dimensions are shown, and the N-terminal helix present in D13^D513G^ is colored magenta. The black triangle denotes the molecular 3-fold symmetry axis. See also Figure S1.

The D13 subunit is composed of two β barrel jelly-roll domains (Figure 1A), V1 (residues 32–223) and V2 (residues 224–262 and 440–547), plus a “turret” domain (T, residues 263–439), an elaboration of a loop of the V2 domain (discussed below). The β barrels follow the jelly-roll topology seen in the capsid proteins of dsDNA viruses such as adenovirus (Athappilly et al., 1994; Rux et al., 2003), PRD1 (Benson et al., 1999, 2002) and PBCV-1 (Nandhagopal et al., 2002). Each barrel contains eight antiparallel β strands (B_1_–I_1_ and B_2_–I_2_, respectively) arranged into two sheets (strands B-I-D-G and C-H-E-F, respectively) (Figure 1A). The network of loops connecting the β strands form distinctive projections above each barrel, described as “towers” in other viral jelly-roll structures (Benson et al., 2004) and harbor several helices and short β strands. The F_1_-G_1_ loop contains an eight-residue helix (α_1_FG_1_) that bisects the V1 and V2 barrels. A similar helix (αFG_2_) sits in the loop connecting the F_2_ and G_2_ strands of the V2 jelly-roll. Both helices lie almost orthogonal to the jelly-rolls, defining the relative orientation of the barrels with respect to each other in the trimer. The loops between β strands D_1_-E_1_, F_1_-G_1_, and H_1_-I_1_ pack tightly together and possess short β strands βDE_1_, βFG_1_, and βHI_1_ that form a three-stranded sheet, at the top of the V1 tower.

Overall the D13^D513G^ mutant is almost identical to the wild-type protein (0.3 Å root-mean-square deviation [rmsd] over 533 C^α^ atoms). The D513G mutation lies in the H_2_-I_2_ loop (Figures 1A and 1B) and the glycine introduces a degree of flexibility to the loop, which adopts three slightly different conformations in the subunits of the D13^D513G^ trimer. In addition, the mutant structure contains well-resolved electron density for residues 2–10 at the N terminus of the protein (not observed in the wild-type structure), which form a short α helix that precedes the long N-terminal tail of each subunit (Figures 1A and 1B).

### Comparison of D13 to the Major Coat Proteins of Other dsDNA Viruses

The architecture of the D13 jelly-rolls is clearly related to the capsid proteins of adenovirus, PBCV-1, STIV, PRD1, and PM2, which based on structural similarity have been grouped into a single “PRD1-adenovirus” lineage (Abrescia et al., 2008; Bamford et al., 2005). These capsid proteins and D13 share double barrel domains with identical topology and major insertions in corresponding loops (the D-E, F-G and H-I loops) (Benson et al., 2004; Krupovic and Bamford, 2008). Structural superposition reveals the PBCV-1 Vp54 capsid protein to be the most similar to D13, aligning 303 C^α^ residues (57% of D13 and 73% of Vp54) with an rmsd of 2.3 Å.

**Figure 2:**
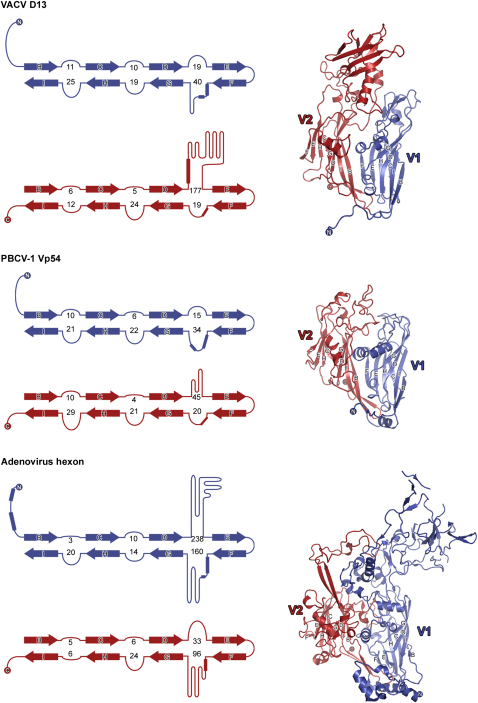
Comparison of VACV D13, PBCV-1 Vp54, and Adenovirus Hexon Capsid Proteins Topology diagrams (left) depict the arrangement of the β strands (arrows) and certain α helices (rectangles) of each jelly-roll. V1 (N-terminal) and V2 (C-terminal) jelly-rolls are colored blue and red respectively. The numbers of residues present in the loops connecting the β strands are indicated. Ribbon diagrams of D13, Vp54, and hexon are shown on the right with the BIDG and CHEF β sheets labeled for each jelly-roll. The N- and C-termini are shown as spheres and labeled. See also Table S1.

The most striking difference between D13 and the MCPs of the PRD1-adenovirus lineage lies in the unprecedented extent of the D_2_-E_2_ loop in D13, which forms a separate domain of 177 residues (263–439) (Figure 2). This turret is visible even in the low resolution electron microscopy reconstruction of the Orf virus ortholog (Hyun et al., 2007). The chain extending from β strand D_2_ forms a 16 residue long helix (residues 280–295): the core of the turret. The C terminus of this helix is flanked on one side by a β sheet formed by strands Tβ4, Tβ6, and Tβ7, and on the other by a short helix (Tα5) and the Tβ1-Tβ2 loop (see Figure S1 available online). The H_2_-I_2_ loop projects 17 Å vertically from V2, forming a buttress that supports the turret domain via contact with the Tβ5-Tβ6 loop. In contrast the insertions between the D_2_ and E_2_ strands in related proteins are shorter (Figure 2). PBCV-1 Vp54, the closest structural homolog to D13, contains an insertion of 45 residues at this point (Nandhagopal et al., 2002), adenovirus hexon 30 residues, and the MCPs of PM2, PRD1, and STIV between 3 and 10 residues. SSM (Krissinel and Henrick, 2004) does not identify any significant structural homologs of the turret (no Z scores >1.0). The functional role of the turret is unknown, although it is well placed to be involved in interactions on the outside of the IV particle.

### D13 Trimer Structure

The architecture of the D13 trimers is similar to that of others of the PRD1-adenovirus lineage (Figure 1B). The trimer is ∼89 Å in diameter at its widest point and 96 Å high, second in size only to the adenovirus hexon, which stands 113 Å tall due to the extensive towers formed from loops of the V1 jelly-roll (superposition of D13 with the adenovirus hexon gives 243 C^α^ equivalences with 3.2 Å rmsd) (Athappilly et al., 1994) (Figure 2). V1 and V2 jelly-rolls alternate around the molecular 3-fold axis, lending a pseudo-hexagonal symmetry to the lower part of the trimer. The packing of adjacent β barrels is stabilized by complementary electrostatic charges; the edge of the V1 jelly-roll and the V1 tower present negatively charged residues that interact with a positively charged patch at the edge of the V2 jelly-roll. In contrast the three turret domains interact to form a cage that gives a triangular shape to the top of the trimer (Figure 1B). The trimerization interface is completed by the N-terminal tail of each subunit (residues 15–31) that extends 43 Å from the V1 jelly-roll and wraps underneath the neighboring subunits (Figure 1B) locking the base of the trimer together. Analysis of the packing interface between subunits (PISA) (Krissinel and Henrick, 2007) confirms that the trimerization interface is the only significant interaction surface in the crystal, with 4220 Å^2^ of occluded surface between adjacent subunits. In total, therefore, 12,660 Å^2^ of surface is buried through trimerization, 16% of the total surface area of the trimer. D13^D513G^ and wild-type D13 trimers differ only in the additional short N-terminal helix (residues 2–10) visualized in the structure of D13^D513G^. This “hook” projects upward from the trimer base and is wedged between the V1 and V2 jelly-rolls of the other two adjacent subunits in the trimer (Figure 1B), contributing an extra 1152 Å^2^ of buried surface area to that subunit interface. The mutated residue, 513 lies in a solvent-accessible position on the outside of the trimer.

## Discussion

**Figure 3:**
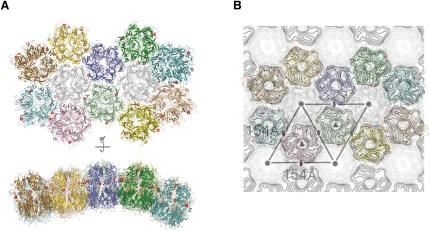
Structural Model of Trimer-Trimer Interactions in the D13 Lattice VACV D13 trimers were superposed using SHP (Stuart et al., 1979) onto a facet of P3 capsid proteins from the related dsDNA virus PRD1. (A) The lattice is viewed, in the top, from the membrane looking up with a view perpendicular to the membrane (out of the virus particle), whereas the bottom shows the view tangential to the viral crescent membrane (note the curvature of the IV particle will be somewhat less, because it is larger than PRD1). Individual trimers are colored separately, and the position of the D513G mutation is shown with a red sphere. Two D13 trimers are drawn semitransparent and colored gray, to show the positions of systematic absences of trimers in the VACV IV lattice that give rise to the honeycomb structure. (B) The D13 trimers from (A) are superimposed onto the projection density of the orfv075 lattice II, which is taken to be a model for the honeycomb lattice seen on the IV particles (Figure 4B of Hyun et al., 2007, reproduced with permission from the *Journal of Virology*).

Crescent structures reminiscent of those formed by D13, but much smaller, have been observed recently for STIV (Fu et al., 2010). Given the strong structural similarity of D13 to the MCPs of viruses of the PRD1-adenovirus lineage we have attempted to use the high resolution information available for PRD1 to construct a plausible model for the organization of the honeycomb structure of the IV particle. We have done this by simply superposing D13 onto appropriate trimers within the surface lattice of PRD1. This seems a reasonable approach because the topology of trimer-trimer interactions is broadly conserved across the four viruses of this lineage for which a reliable atomic or quasi-atomic model can be constructed. The spacing of trimers in the modeled D13 lattice is such that plausible contacts are formed between adjacent trimers and the lattice geometry is consistent with that observed by electron microscopy for 2-D crystals of the orthologous protein from Orf virus (Figure 3) (center-to-center spacing ∼90 Å) (Hyun et al., 2007). The orientation of the trimers within the PRD1-derived lattice matches the electron microscopy data and the morphology-modifying residue 513 lies at the interface between neighboring trimers (Figure 3). Despite the striking similarity between the organization of the surface lattice of D13 and other viruses of the PRD1-adenovirus lineage there remains a fundamental difference because D13 possesses only two-thirds of the complement of trimeric molecules (Figure 3) leading to the characteristic honeycomb arrangement. We propose that this honeycomb lattice characterizes a clade within the established PRD1-adenovirus lineage, which may also include the giant mimivirus (Xiao et al., 2009).

**Figure 4:**
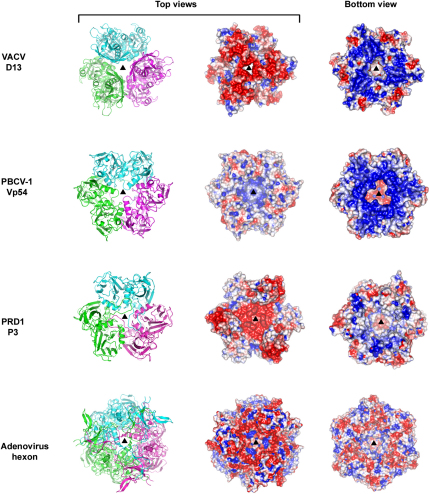
Comparison of Surface Charge Distribution on dsDNA Virus MCPs Trimers of D13, Vp54, P3, and hexon are shown as cartoons (left, each subunit colored separately) and electrostatic surfaces (middle and right) and viewed from the top and bottom along their 3-fold symmetry axes (black triangles). Electrostatic charges are contoured from red (−3 *k*T/e) to blue (+3 *k*T/e).

Based on the model for the honeycomb surface lattice of the IV particles presented in Figure 3, we can estimate the number of D13 subunits per particle. Given that the average radius (at the base of the D13 trimer) for an IV particle is some 1500 Å, there are likely to be ∼5500 trimers on each particle. These numbers make the IV particle one of the largest established members of the adeno-PRD1 lineage, although we cannot be certain that all IV particles possess the same number of D13 subunits. The lattice model also predicts the orientation of the D13 trimer with respect to the membrane. In agreement with this the trimer displays a polarized charge distribution over its surface (Figure 4). The triangular cage formed by the turret domains presents a negatively charged outer surface at the top of the trimer (Figure 4), whereas the N-terminal subunit tails form a positively charged surface at the base of the trimer close to the negatively charged membrane (Figure 4). This will facilitate correct orientation of the D13 trimers during scaffold formation on the viral crescent membranes. The calculated pI of D13 is 5.4, identical to both PRD1 P3 and adenovirus hexon capsid proteins. In both D13 and PRD1 the negative charge is localized distal to the viral membrane whereas in adenovirus, which does not contain a membrane, negatively charged surfaces are present at both poles of the trimer. Although Vp54 of PBCV-1 has a pI of 7.8, it has a positively charged surface at the trimer base (Figure 4) adjacent to the internal viral membrane. In addition to these electrostatic characteristics, the somewhat separate amphipathic N-terminal helix of D13 (residues 2–10, seen in the D13^D513G^ structure) may also stabilize interaction with the membrane by more direct contacts. D13 is not an integral membrane protein and an interaction has been reported between D13 and the N terminus of the VACV integral membrane protein A17 (Bisht et al., 2009), which is crucial for VACV crescent formation (Wolffe et al., 1996). The loosely attached N-terminal helix of D13 is well placed— at the base of the trimer—to form possible interactions with A17 and the membrane. The lack of well-defined density for the intervening residues (11–14) between the N-terminal helix and the N-terminal tail suggest a possible flexible linker that would facilitate the movement of this helix during complex formation with A17 and possibly other VACV crescent membrane proteins. Such interactions may be analogous to those observed between the PRD1 and PM2 capsid proteins and the minor proteins in these bacteriophages that define the size of the icosahedral facets (Abrescia et al., 2004, 2008).

Despite concerted efforts, we were unable to visualize rifampicin in D13 crystals that had been soaked with or crystallized in the presence of the drug (data not shown). VACV mutations, giving resistance to rifampicin, map to the *D13L* gene (Baldick and Moss, 1987; McNulty-Kowalczyk and Paoletti, 1993) and random PCR mutagenesis has identified 24 resistance mutations (Charity et al., 2007). Of these. nine are localized at the base of the D13 trimer (Figure S2). The remainder are close to the trimer base and are solvent accessible. The majority of the mutations increase the hydrophobic character of D13, which may allow D13 to overcome the disruption rifampicin has on membrane association. We suggest that rifampicin acts by direct (but nonspecific) interactions with D13 or by membrane-mediated interactions to reduce the affinity of D13 for the IV membrane.

**Figure 5:**
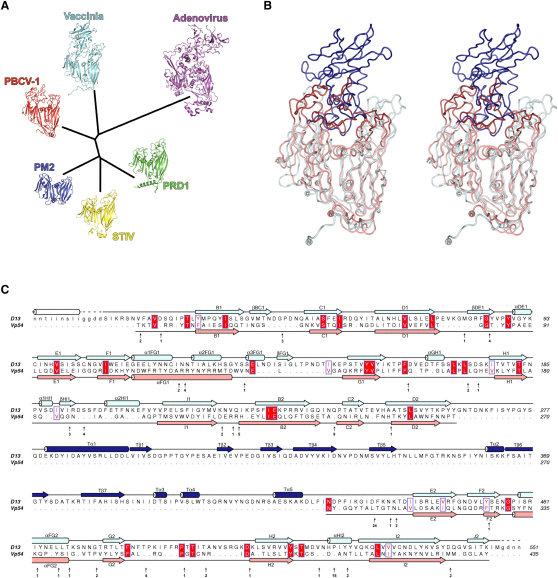
Structure-Based Phylogenetic Tree of the PRD1-Adenovirus Lineage and Comparison of VACV D13 to PBCV-1 Vp54 (A) Structures of capsid proteins from vaccinia (D13), PBCV-1 (Nandhagopal et al., 2002), adenovirus (Rux et al., 2003), STIV (Khayat et al., 2005), PRD1 (Benson et al., 2002), and PM2 (Abrescia et al., 2008) were superposed and a pairwise distance matrix was constructed as described previously (Bamford et al., 2005; Riffel et al., 2002). Cartoons of each capsid protein are drawn at the branch ends, colored separately and labeled with their virus names. (B) Stereo view of VACV D13 and PBCV-1 Vp54 superposed by their Cα traces, with their core jelly-roll domains colored light blue and salmon, and the turret domain and tower loops dark blue and red, respectively. N- and C-termini are shown as spheres and labeled. (C) Structure-based sequence alignment of VACV D13 and PBCV-1 Vp54 derived from the superposition via SHP (Stuart et al., 1979). The secondary structures of D13 and Vp54 are drawn above and below the alignment respectively, and colored as in (B); β strands are shown as arrows and α helices as cylinders. The N-terminal helix seen in D13^D513G^ is drawn as a white cylinder. Strictly conserved residues are boxed in red, moderately conserved residues are boxed white with a red face, and conservation is scored according to the Blosum62 scoring matrix. Residues of Vp54 that are not aligned by the structural superposition are omitted and their number and positions are indicated under the Vp54 sequence with black arrows. See also Table S1.

D13 displays clear structural homology with the capsid proteins of the PRD1-adenovirus lineage (Krupovic and Bamford, 2008) that extends beyond the core architecture of a double β barrel, despite having no sequence similarity (the greatest sequence identity for structurally equivalent residues is 10% for Vp54) (Table S1). The greatest difference between D13 and other members of the lineage is the presence of the turret domain, which is formed from a single insertion (between strands D_2_ and E_2_) and is quite different in topology from all other known structures. Although the fold of the turret domain is unique to D13 the presence of elaborations in the “top” (membrane distal) portion of the molecule is a general feature of the molecules from viruses infecting higher eukaryotes (Figure 2). To systematically compare D13 to the capsid proteins of this virus lineage a structure-based phylogenetic tree was calculated (Bamford et al., 2005) using pairwise structural superpositions of D13 and the capsid proteins from PRD1, PM2, STIV, PBCV-1, and adenovirus (Figures 5A–5C). The overall topology of the phylogenetic tree from this analysis follows that seen before (Abrescia et al., 2008), with the capsid proteins from STIV and the bacteriophages PRD1 and PM2 all clustering separately from Vp54, D13, and the adenovirus hexon. The capsid proteins from viruses that infect higher eukaryotes have diverged substantially from those of the bacteriophages and archaea, due to progressively larger tower loops above the V1 or V2 jelly-rolls. D13 fits comfortably within this lineage, the most divergent structure being the adenovirus hexon (reflecting the fact that this virus does not contain a membrane). That D13 is well embedded within this lineage is perhaps surprising because mature vaccinia virus is the only currently identified member of this lineage that does not possess icosahedral symmetry. This further strengthens the argument that this structural lineage is extraordinarily well-suited to the assembly of very large virus particles and is widely used across all three domains of life. Most remarkably the structure of VACV D13 and its role in the construction of a spherical immature virion suggests that the morphogenesis of complex viruses, in a manner analogous to embryogenesis of higher organisms, can recapitulate their evolutionary history and it will be interesting to see if this principle can be used to locate other complex, non-icosahedral viruses within established viral lineages.

## Experimental Procedures

### Infusion Cloning

Full-length wild-type D13 (residues 1–551) was amplified by PCR from VACV Western Reserve cDNA using KOD HiFi DNA polymerase (Novagen) according to the manufacturer's instructions (forward and reverse primers: 5′-AAGTTCTGTTTCAGGGCCCGAATAATACTATCATTAATTCTTTGATCG-3′ and 5′-ATGGTCTAGAAAGCTTTAGTTATTATCTCCCATAATCTTGGTAA-3′, respectively). The gene was cloned into expression plasmid pOPINF (adding an N-terminal His_6_ fusion tag and a rhinovirus 3C protease cleavage site) by InFusion cloning (Berrow et al., 2007), and the sequence verified.

### Site-Directed Mutagenesis

An A to G point mutation at nucleotide position 1538 of the *D13L* gene was introduced using the QuikChange site-directed mutagenesis kit (Stratagene) (forward and reverse primers: 5′-GAGTAGTTTATTCCACCATGGGTGTCAACCATCCAATCTATTA-3′, 5′-TAATAGATTGGATGGTTGACACCCATGGTGGAATAAACTACTC-3′, respectively), according to the manufacturer's instructions, producing a D13^D513G^ mutant. The final plasmid was sequence verified.

### Large-Scale Expression

Unlabeled D13 and selenomethionine (SeMet)-labeled D13^D513G^ were overexpressed in *E. coli* B834(DE3) (grown in the presence of 50 μg/ml carbenicillin) by autoinduction (Studier, 2005). For unlabeled D13, cells were grown in Overnight Express Terrific Broth (Novagen). For SeMet-labeled D13^D513G^, cells were grown in glucose-free SeMet medium (Molecular Dimensions) supplemented with Overnight Express Autoinduction System 1 (Novagen) and SeMet (90 μg/ml). All cell cultures were grown at 37°C for ∼6 hr, 225 rpm, cooled to 25°C, allowed to grow for a further 20 hr before harvesting by centrifugation (6000 g at 8°C for 20 min) and stored frozen (−80°C) until required.

### Purification of Native and SeMet-Labeled D13

Frozen cell pellets were thawed and resuspended into 50 mM Tris pH 7.5, 500 mM NaCl, 50 mM imidazole, 0.2% (v/v) Tween-20, supplemented with 400 units of bovine pancreas deoxyribonuclease 1 (Sigma) and 2 EDTA-free protease inhibitor tablets (Roche). Cells were lysed using a Basic Z model cell disruptor (Constant Systems) at 30 kPa. The lysate was cleared by centrifugation (35,000 g, 8°C, 30 min) and proteins were purified by Ni-NTA affinity chromatography followed by size exclusion chromatography on a HiLoad 16/60 Superdex 200 gel filtration column (GE Healthcare) pre-equilibrated in 20 mM Tris pH 7.5, 300 mM NaCl and 2 mM TCEP. SDS-PAGE confirmed the purity (>98%) of the protein and mass-spectrometry confirmed both the identity of unlabeled D13 and the percentage Se incorporation (100%) of SeMet-labeled D13^D513G^ (data not shown).

### Crystallization and Data Collection

Purified samples of wild-type D13 and D13^D513G^ were concentrated to 5.5 mg/ml and 3.3 mg/ml, respectively, using 30 kDa MWCO microconcentrators (Amicon). Initial screening of crystallization conditions was performed at 21°C by vapor diffusion in 96-well sitting-drop plates (Greiner) containing 100 nL protein + 100 nL reservoir equilibrated against 95 μl of reservoir solution (Brown et al., 2003; Walter et al., 2003). D13 and D13^D513G^ crystallized readily against reservoir containing 3.5–4.0 M sodium formate (∼pH 7.0), and initial crystals were optimized by varying the pH, drop ratio of protein to reservoir and dilutions of the reservoir concentration (Walter et al., 2005). Diffraction quality crystals belonging to space group *P*6_1_22 appeared within 24 hr for both protein forms. Crystals of D13^D513G^ were mounted from the sodium formate mother liquor and immediately flash frozen. For wild-type D13, superior diffraction data were collected from crystals passed through reservoir solution supplemented with 20% v/v glycerol before flash-freezing. Diffraction data were collected at 100 K at the Diamond synchrotron beamline IO3 (Table 1) and analyzed with XDS (Kabsch, 1993) and SCALA (Evans, 2006) (implemented in the program XIA2 [Winter, 2010]).

### Structure Solution, Refinement, and Validation

A single-wavelength anomalous dispersion (SAD) experiment allowed the positions of the 24 selenium atoms in the D13 trimer to be located, refined, and phases calculated with SHELXD (Schneider and Sheldrick, 2002) and SHARP (de la Fortelle and Bricogne, 1997) (using AutoSHARP [Vonrhein et al., 2007]). Solvent flattening was performed with DM (Cowtan, 1994) and SOLOMON (Abrahams and Leslie, 1996). Three-fold noncrystallographic symmetry (NCS) averaging using GAP (unpublished program) produced an excellent electron density map, allowing the bulk of the backbone chain to be traced manually and then finalized with CALPHA (Esnouf, 1997). The higher resolution (2.8 Å) structure of wild-type D13 was determined directly from the isomorphous structure solved by SAD analysis of D13^D513G^. Manual model building was performed using COOT (Emsley et al., 2010). Refinement for both wild-type D13 and D13^D513G^ was initially performed with phenix.refine (Afonine et al., 2005) and then BUSTER-TNT (Bricogne et al., 2010); imposing 3-fold NCS restraints throughout refinement (Smart et al., 2008), in consultation with the validation tools present in COOT and MolProbity (Davis et al., 2007). Refinement statistics are shown in Table 1.

### Structural Analysis

A representative set of capsid protein structures used for structure-based phylogenetic analysis were selected with the use of the Secondary Structure Matching (SSM) web server (Krissinel and Henrick, 2004). For structures where more than two capsid proteins were present in the asymmetric unit the most representative monomer was determined with the MCentral command of LSQMAN (Kleywegt and Jones, 1997) and then used for analysis. A gap-penalty-weighted superposition of all capsid proteins was performed with a version of the program SHP (Stuart et al., 1979), modified to estimate the evolutionary distance (Bamford et al., 2005; Riffel et al., 2002). A full matrix of evolutionary distances was calculated and the tree representation was generated from this distance matrix, using the programs FITCH and DRAWTREE, as part of the PHYLIP package (Feisenstein, 1989) with default parameters. Data used to generate the phylogenetic tree are presented in Table S1.

Structure-based multiple sequence alignments were generated from superposed coordinate files using shp2clustalw (unpublished program) and edited using ALINE (Bond and Schuttelkopf, 2009). Electrostatic potentials were calculated using APBS (Baker et al., 2001) and pdb2pqr (Dolinsky et al., 2007). Figures were rendered using PyMOL (DeLano, 2008).

## Figures and Tables

**Table 1:** Data Collection and Refinement Statistics

	SeMet D13^D513G^	Unlabeled D13 wild-type
**Data collection statistics**	Se-SAD Peak	High resolution native
Beamline	Diamond IO3	Diamond IO3
Wavelength (Å)	0.9792	0.9782
Resolution limits (Å)^a^	39.3–3.0 (3.1–3.0)	46.2–2.8 (2.9–2.8)
Space group	*P*6_1_22	*P*6_1_22
Unit cell dimensions (Å)	a = b = 190.6 c = 253.0	a = b = 190.1 c = 257.1
Unique reflections^a^	53,583 (3818)	64,722 (4681)
Redundancy^a^	20.8 (15.4)	11.0 (10.9)
Completeness (%)^a^	99.7 (98.6)	96.9 (95.5)
<*I*/σ(*I*) >^a^	16.9 (2.0)	19.0 (2.7)
*R*_merge_a,b	0.21 (1.5)	0.09 (0.97)
**Refinement statistics**		
Resolution limits (Å)^a^	39.3–3.0 (3.1–3.0)	46.2–2.8 (2.9–2.8)
Number of reflections in working set^a^	50,749 (3625)	61,439 (4411)
Number of reflections in test set^a^	2713 (182)	3281 (263)
*R*_xpct_a,c	0.170 (0.243)	0.177 (0.250)
*R*_free_a,c,d	0.203 (0.298)	0.206 (0.295)
Number of atoms (protein/water)	12,913/79	12,717/147
Number of atoms with alternate conformations (protein/water)	13/0	8/0
Residues in Ramachandran favored region (%)	95.6	95.4
Ramachandran outliers (%)	0.0	0.0
rmsd^e^ bond lengths (Å)	0.009	0.009
rmsd^e^ bond angles (°)	1.100	1.040
Average *B* factors (protein/water) (Å^2^)	62.4/44.2	67.2/61.3

a Numbers in parentheses refer to the appropriate outer shell.

b *R*_merge_ = Σ_hkl_ Σ_i_|*I*(*hkl;i*) – <*I*(*hkl*)> |/Σ_hkl_ Σ_i_*I*(*hkl;i*), where *I*(*hkl;i*) is the intensity of an individual measurement of a reflection and <*I*(*hkl*)> is the average intensity of that reflection.

c *R*_xpct_ = Σ_hkl_‖*F*_obs_| –|*F*_xpct_‖/Σ_hkl_ |*F*_obs_|, where |*F*_obs_| and |*F*_xpct_| are the observed structure factor amplitude and the expectation of the model structure factor amplitude respectively.

d *R*_free_ equals the *R*_xpct_ of test set (5% of the data removed prior to refinement).

e rmsd, root-mean-square deviation from ideal geometry.

## Accession Numbers

Atomic coordinates and structure factors have been deposited with the Worldwide Protein Data Bank, accession codes 2ygb (wild-type D13) and 2ygc (D13^D513G^).

## Supplemental Information

Document S1. Two Figures and One Table
