# Supplementary material for: Insights into the Evolution of a Complex Virus from the Crystal Structure of Vaccinia Virus D13
Source: Structure. 2011 Jul 13;19(7-12):1011–20. doi: 10.1016/j.str.2011.03.023 (PMC3136756; doi:10.1016/j.str.2011.03.023)
Supplement: Document S1. Two Figures and One Table [file mmc1.pdf]

Structure *19*

Supplemental Information

Insights into the Evolution of a Complex Virus from the Crystal Structure of Vaccinia Virus D13

Mohammad W. Bahar, Stephen C. Graham, David I. Stuart, and Jonathan M. Grimes

Table S1, related to Figure 2 and Figure 5. Structural and sequence similarity of dsDNA virus capsid proteins belonging to the PRD1-adenovirus lineage. Names of each capsid protein and their PDB IDs are shown. Cα rmsds and number of equivalent residues (in parentheses) are shown above the diagonal, evolutionary distances and sequence identities of equivalent residues (%, in parentheses) are shown below.

	VACV D13 (xxxx)	PM2 P2 (2VVF)	PRD1 P3 (1HX6)	STIV MCP (2BBD)	PBCV-1 Vp54 (1M3Y)	Adenovirus Hexon (1P30)
VACV D13 (xxxx)	-	2.89 (213)	3.15 (270)	3.02 (248)	2.30 (306)	3.30 (248)
PM2 P2 (2VVF)	1.83 (9.4)	-	2.47 (231)	2.32 (226)	2.68 (229)	3.16 (125)
PRD1 P3 (1HX6)	1.78 (9.3)	1.39 (8.2)	-	2.18 (278)	3.02 (271)	3.60 (192)
STIV MCP (2BBD)	1.77 (7.7)	1.26 (9.7)	1.22 (11.9)	-	2.70 (257)	3.44 (147)
PBCV-1 Vp54 (1M3Y)	1.43 (9.8)	1.51 (12.7)	1.61 (8.9)	1.48 (10.5)	-	3.44 (255)
Adenovirus Hexon (1P30)	2.40 (7.3)	2.86 (11.2)	2.65 (3.1)	2.69 (1.4)	2.32 (6.7)	-

Figure S1.

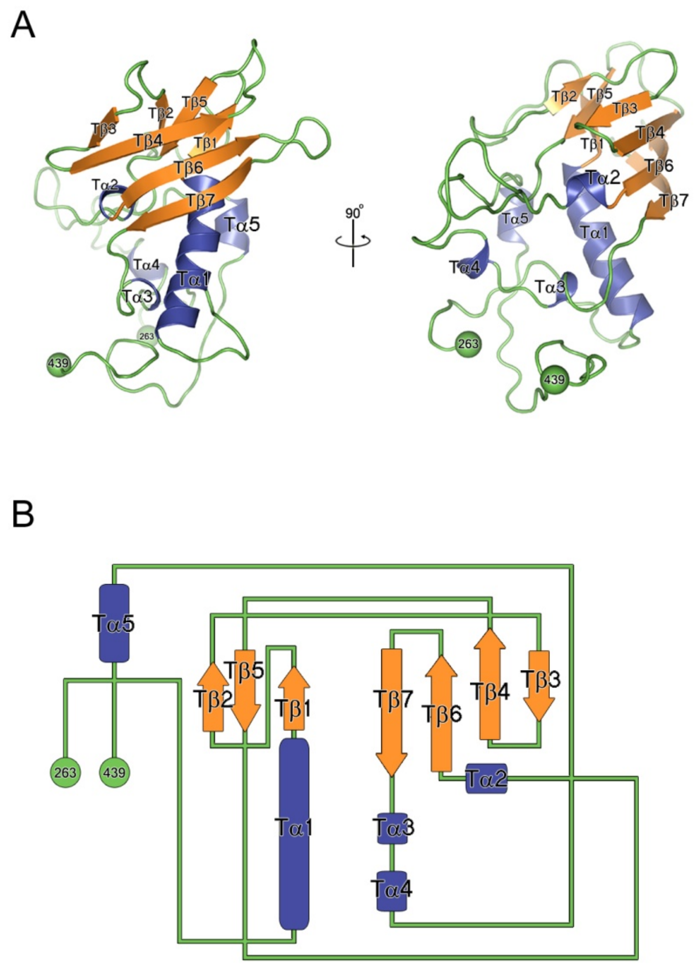

Figure S1, related to Figure 1. Structure and topology of the D13 turret domain. (**A**) D13 turret domain is shown as a ribbon, with α helices coloured blue, β strands orange and connective loops in green. Green spheres denote the start and end residues of the turret domain and are numbered. (**B**) Topology diagram of the turret domain, coloured and labelled as in **A**.

Figure S2.

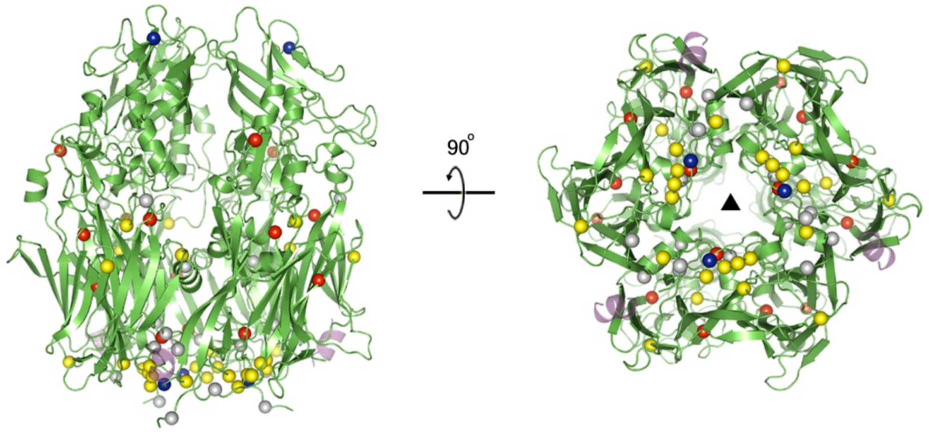

Figure S2. Distribution of rifampicin resistant mutations on the D13 trimer. A trimer of D13 is shown as ribbons, coloured green and viewed from the side (left) and from the base (right). The N-terminal helix present in the $D13^{D513G}$ structure is shown as a semi-transparent magenta ribbon. Positions of rifampicin resistance mutations reported by Charity et al. (2007) are shown as spheres and coloured according to their chemical property as red (acidic), blue (basic), yellow (hydrophobic) and grey (neutral).
